# Improving Accuracy of Influenza-Associated Hospitalization Rate Estimates

**DOI:** 10.3201/eid2109.141665

**Published:** 2015-09

**Authors:** Alexander J. Millman, Carrie Reed, Pam Daily Kirley, Deborah Aragon, James Meek, Monica M. Farley, Patricia Ryan, Jim Collins, Ruth Lynfield, Joan Baumbach, Shelley Zansky, Nancy M. Bennett, Brian Fowler, Ann Thomas, Mary L. Lindegren, Annette Atkinson, Lyn Finelli, Sandra S. Chaves

**Affiliations:** Centers for Disease Control and Prevention, Atlanta, Georgia, USA (A.J. Millman, C. Reed, L. Finelli, S.S. Chaves);; California Emerging Infections Program, Oakland, California, USA (P. Daily Kirley);; Colorado Department of Public Health and Environment, Denver, Colorado, USA (D. Aragon);; Connecticut Emerging Infections Program, New Haven, Connecticut, USA (J. Meek);; Emory University School of Medicine, Atlanta (M.M. Farley);; Atlanta Veterans Administration Medical Center, Atlanta (M.M. Farley);; Maryland Department of Health and Mental Hygiene, Baltimore, Maryland, USA (P. Ryan);; Michigan Department of Health and Human Services, Lansing, Michigan, USA (J. Collins);; Minnesota Department of Health, St. Paul, Minnesota, USA (R. Lynfield);; New Mexico Department of Health, Santa Fe, New Mexico, USA (J. Baumbach);; New York State Department of Health, Albany, New York, USA (S. Zansky);; University of Rochester School of Medicine and Dentistry, Rochester, New York, USA (N.M. Bennett);; Monroe County Department of Public Health, Rochester (N.M. Bennett);; Ohio Department of Health, Columbus, Ohio, USA (B. Fowler);; Oregon Public Health Division, Portland, Oregon, USA (A. Thomas);; Vanderbilt University School of Medicine, Nashville, Tennessee, USA (M.L. Lindegren);; Utah Department of Health, Salt Lake City, Utah, USA (A. Atkinson)

**Keywords:** influenza, hospitalizations, sensitivity, diagnostic tests, viruses

## Abstract

Diagnostic test sensitivity affects rate estimates for laboratory-confirmed influenza–associated hospitalizations. We used data from FluSurv-NET, a national population-based surveillance system for laboratory-confirmed influenza hospitalizations, to capture diagnostic test type by patient age and influenza season. We calculated observed rates by age group and adjusted rates by test sensitivity. Test sensitivity was lowest in adults >65 years of age. For all ages, reverse transcription PCR was the most sensitive test, and use increased from <10% during 2003–2008 to ≈70% during 2009–2013. Observed hospitalization rates per 100,000 persons varied by season: 7.3–50.5 for children <18 years of age, 3.0–30.3 for adults 18–64 years, and 13.6–181.8 for adults >65 years. After 2009, hospitalization rates adjusted by test sensitivity were ≈15% higher for children <18 years, ≈20% higher for adults 18–64 years, and ≈55% for adults >65 years of age. Test sensitivity adjustments improve the accuracy of hospitalization rate estimates.

## Abstract 2

Adjusting for diagnostic test sensitivity enables more accurate and timely comparisons over time.

In the United States, surveillance for influenza-associated hospitalizations relies on laboratory-confirmed diagnostic testing (*1*–*3*). Influenza testing modalities have expanded from traditional viral culture to include rapid influenza diagnostic tests (RIDTs) and molecular assays, such as reverse transcription PCR (RT-PCR) (*4*,*5*). RIDTs are point-of-care tests that provide results within 30 minutes; however, with reported sensitivities of 10%–80%, negative test results can be unreliable (*6*–*9*). RT-PCR exceeds viral culture in sensitivity for detecting influenza, but its widespread use is limited by cost and complexity of the assay (*10*,*11*).

Researchers have examined rates of influenza-associated hospitalization during different influenza seasons (*1*,*2*,*12*,*13*). However, comparing rates between seasons can be inaccurate without accounting for changes in the sensitivity of diagnostic testing used. In particular, after the 2009 influenza A(H1N1) pandemic, hospitals and state public health laboratories expanded diagnostic capabilities with high-sensitivity molecular assays to better detect influenza viruses and other respiratory pathogens (*5*). Particularly for nationally based surveillance, the use of different testing platforms by health care facilities and the variability in sensitivity of these diagnostic tests could lead to underestimation of rates of influenza-associated hospitalization and limit comparisons of severity across influenza seasons (*3*,*4*,*6*,*7*).

## Methods

### Study Setting

We used data from the Centers for Disease Control and Prevention (CDC) Influenza Hospital Surveillance Network (FluSurv-NET) from the 2003–2013 influenza seasons (*3*,*14*). FluSurv-NET conducts population-based surveillance for laboratory-confirmed influenza-associated hospitalizations among children <18 years of age (since the 2003–04 influenza season) and adults (since the 2005–06 influenza season). The FluSurv-NET system and protocol have been described previously (Technical Appendix) (*1*,*3*,*15*).

CDC determined that data collected through FluSurv-NET were for routine public health surveillance and not subject to institutional review board approval for human research protections. Participating sites submitted the surveillance protocol to their state and local institutional review boards for review.

### Case Definition and Data Collection

A case of influenza-associated hospitalization was defined as hospitalization of a catchment-area resident who was hospitalized in a catchment-area hospital during a designated influenza season (October 1–April 30) with a laboratory-confirmed influenza test within 14 days before or 3 days after hospital admission. Laboratory-confirmed influenza was defined as a positive result from RT-PCR, viral culture, direct fluorescent antibody staining (DFA), or RIDT or a positive result for an unspecified laboratory test documented in the medical chart. RT-PCR could be performed at the participating hospital or at the state public health laboratory depending on test availability in the hospital laboratory. The frequency of identified cases by diagnostic test type (observed case count) by patient age and by influenza season was evaluated. When an identified case had >1 type of positive influenza test, we used the test type with the highest sensitivity—RT-PCR, viral culture, DFA, RIDT (ordered from highest to lowest sensitivity)—for the analysis. If an identified case had no other test type and a positive result from an unspecified laboratory test documented in the medical chart, we assumed 100% sensitivity for that test.

### Diagnostic Test Sensitivity

We reviewed the literature to obtain sensitivity ranges for influenza diagnostic tests. We searched PubMed with a strategy containing search terms for influenza disease or virus combined with search terms for RT-PCR, viral culture, DFA, and RIDTs and search terms for sensitivity. Search terms for influenza were as follows: “influenza, human” [Medical Subject Heading (MeSh)] OR “influenza A virus” [MeSh] OR “influenza B virus” [MeSh] OR “influenza” or “flu.” Search terms for the tests included “RT-PCR,” “reverse transcription polymerase chain reaction,” “culture,” direct florescent antibody,” “DFA,” “rapid diagnostic test.” Search terms for clinical sensitivity included “sensitivity,” “test characteristics,” “diagnostic test characteristics,” and “test performance characteristics.” We hand-searched bibliographies of included studies and recent narrative reviews of influenza diagnostic tests for additional relevant studies. We included only studies describing the clinical performance of the different diagnostic test types and did not use the manufacturer’s package insert or subtype-specific assessments. We identified studies describing the clinical sensitivities of different diagnostic test types in the system and focused on the periods before and after the 2009 influenza pandemic. The diagnostic reference standard used in the studies was either viral culture or RT-PCR. The sensitivity of influenza diagnostic tests varies by age because of factors, such as differences in viral shedding (*16*–*19*); therefore, we collected characteristics on each test type by age group (children <18 years, adults 18–64 years, and adults >65 years). Because we categorized the influenza diagnostic test type by method, we preferentially selected studies, such as meta-analyses, that could evaluate multiple brands of a particular influenza diagnostic test. We attempted to select studies based in hospitalized or emergency department settings when available.

We abstracted sensitivity values from the literature by age group as a range of minimum to maximum values or as a point estimate with a 95% CI, depending on how the data were reported (Technical Appendix Table 1). To create a summary empirical distribution across all included studies for each age group and test type, we applied bootstrap techniques (*20*). All ranges were evaluated as a single observation and equally weighted in the analysis. We resampled 1,000 times from each reported distribution of test sensitivity (uniform distribution when only a minimum and maximum sensitivity were reported or a normal distribution when the midpoint and 95% CI were reported). To summarize the resulting empirical distribution, we calculated a median estimate and 95% CI for each diagnostic test type by age group.

### Rate Calculations

We calculated rates of influenza-associated hospitalization per 100,000 population using the National Center for Health Statistics (NCHS) population estimates for the counties in the surveillance catchment area. We calculated observed rates per 100,000 population by age group for each season using the observed case count and dividing it by the NCHS population estimate for that age group and influenza season. To adjust the observed hospitalization rates for test sensitivity, we used the following formula to estimate an adjusted case count by age group for each diagnostic test:

(adjusted case count)_test_ = (observed case count)_test_ × (1/sensitivity_test_)

We calculated the total adjusted case count for a season and age group by summing the test-specific adjusted case counts. Finally, we calculated adjusted rates per 100,000 population by dividing the total adjusted case counts by the NCHS population estimate for that age group and season.

To reflect the previously described distribution of test sensitivity, this series of calculations was performed within the previously described bootstrap for each resampled value of test sensitivity. Reported here are the median estimate and 95% CI for each season and age group. All analyses were performed in SAS version 9.3 (SAS Institute, Cary, NC, USA).

## Results

**Figure 1:**
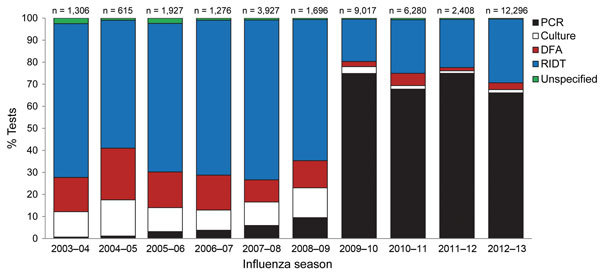
Distribution of influenza diagnostic tests among identified cases in the Centers for Disease Control and Prevention Influenza Hospital Surveillance Network (FluSurv-NET), 2003–2013. RT-PCR, reverse transcription PCR; DFA, direct fluorescent antibody test; RIDT, rapid influenza diagnostic test.

During 2003–2013, the distribution of influenza diagnostic tests among identified cases changed, particularly after the 2009 pandemic (Figure 1). Before 2009, RIDTs were the most common test type, accounting for ≈70% of cases identified in FluSurv-NET. After the 2009 pandemic, RT-PCR became the most frequent test type for all age groups (Technical Appendix Figure). The proportion of RT-PCRs among identified cases increased from <10% before 2009 to ≈70% after 2009.

The Table summarizes the diagnostic test performance characteristics by age group obtained from the literature review and the bootstrap analysis. Influenza diagnostic tests are generally most sensitive when performed on specimens from children <18 years; RT-PCR has the highest sensitivity in this age group (sensitivity estimate 95%, 95% CI 82%–98.7%). The sensitivity of influenza diagnostic tests in adults 18–64 years is similar to that in children <18 years except for RIDTs, which are less sensitive in this age group. Overall, influenza diagnostic tests have poor sensitivity in adults >65 years. RIDTs have the lowest sensitivity in this age group (sensitivity estimate 20.1%, 95% CI 8.8%–41.4%), and although RT-PCR is more sensitive in this age group than are other test types, the midpoint sensitivity estimate for RT-PCR is still <90%. DFA sensitivity appears higher than that of culture and RIDTs in this age group; however, these results were extrapolated from studies that primarily included a younger population (*27*,*28*). Additionally, DFA was seldom performed in this age group (Technical Appendix Table 1).

**Table:** Influenza diagnostic test sensitivity range, by patient age group (years), FluSurv-NET, 2003–2013*

Diagnostic test/patient age group, y	Range from literature review, %	References	Bootstrap estimate (95% CI)
RT-PCR			
0–17	79.2–100	(*19*,*21**–**24*)	95.0 (82–98.7)
18–64	79.2–100	(*19*,*21**–**23*)	94.1 (81.1–98.7)
>65	79.2–93	(*19*,*21*,*25*)	86.1 (79.6–92.7)
Culture			
0–17	45–100	(*4*,*19*,*24*,*26*)	69.3 (48.3–95.9)
18–64	45–100	(*4*,*19*,*26*)	72.8 (47.2–96.3)
>65	19.4–53.8	(*8*,*25*)	36.2 (20.3–52.1)
DFA			
0–17	45–90	(*24*,*27**–**30*)	70.9 (46.8–86.6)
18–64	53–84.2	(*27*,*28*)	68.0 (53.8–83.4)
>65	53–84.2	(*27*,*28*)	68.0 (53.8–83.4)
RIDT			
0–17	61.6–71.7	(*7*)	66.7 (61.3–71.7)
18–64	47.7–59.8	(*7*)	53.9 (47.8–59.8)
>65	8–43	(*8*,*17*,*25*,*31*)	20.1 (8.8–41.4)

*DFA, direct fluorescent antibody; FluSurv-NET, Centers for Disease Control and Prevention Influenza Hospital Surveillance Network; RIDT, rapid influenza diagnostic test; RT-PCR, reverse transcription PCR.

**Figure 2:**
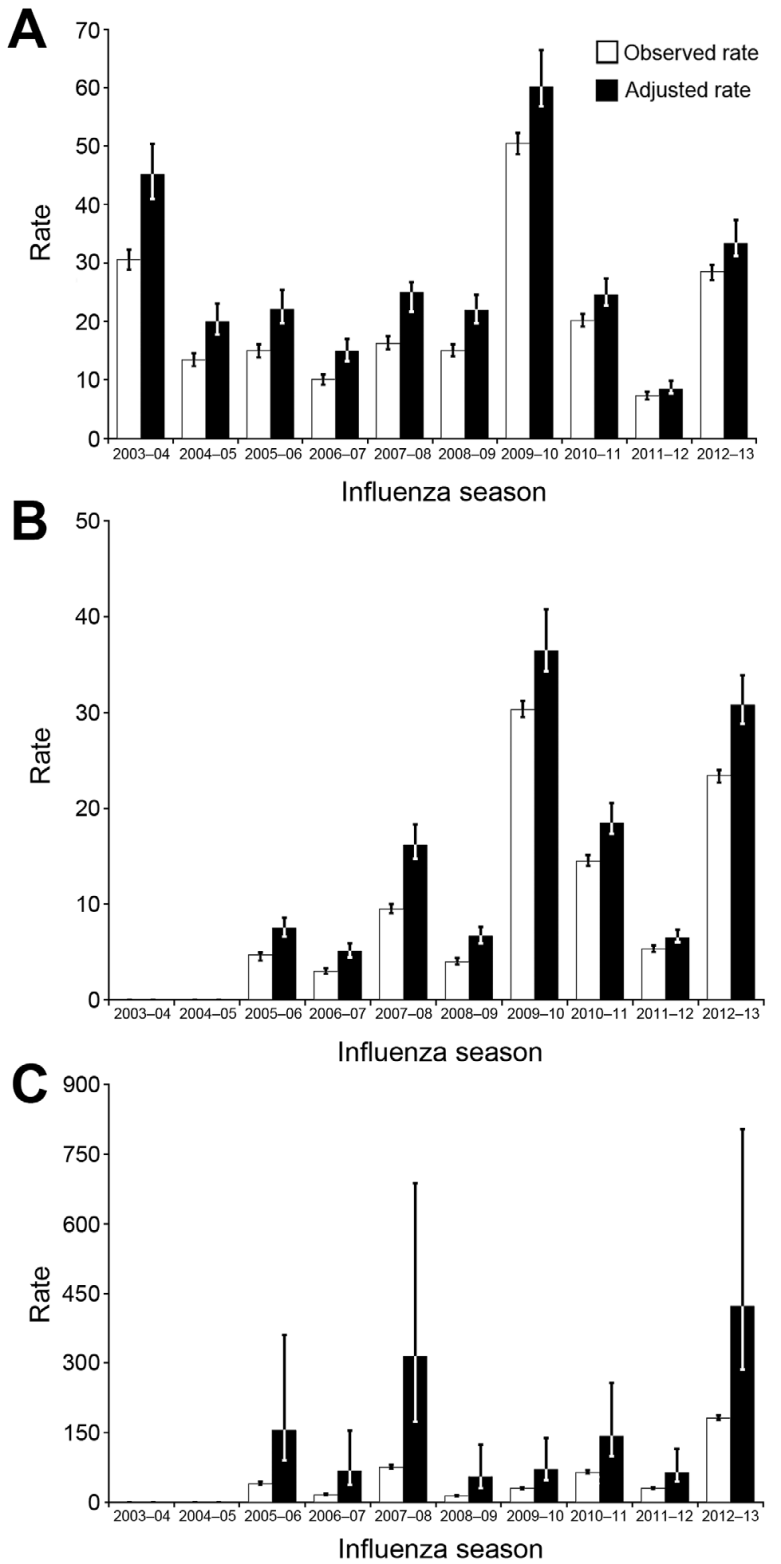
Observed and adjusted rates of influenza-associated hospitalizations per 100,000 population identified in the Centers for Disease Control and Prevention Influenza Hospital Surveillance Network (FluSurv-NET), 2003–2013. A) Children <18 years of age. B) Adults 18–64 years of age. C) Adults >65 years of age. Scale on the y-axis changes for each age group. Error bars indicate 95% CIs.

Observed and adjusted rates of influenza-associated hospitalization per 100,000 population varied by season for all age groups, indicating a particular influenza season’s severity (Figure 2; Technical Appendix Table 2). Observed hospitalization rates ranged from 7.3 during 2011–12 to 50.5 during 2009–10 for children <18 years of age, 3.0 during 2006–07 to 30.3 during 2009–10 for adults 18–64 years, and 13.6 during 2008–09 to 181.8 during 2012–13 for adults >65 years. Hospitalization rates were highest for adults >65 years of age and lowest for adults 18– 64 years of age.

Adjusting for test sensitivity increased hospitalization rates across all age categories (Figure 2). Adjusted rates showed that the number of hospitalizations was higher than previously reported in all seasons for all age groups, regardless of the severity of the season; however, rates increased more in earlier seasons. The magnitude of hospitalizations during severe influenza seasons during earlier surveillance years (2003–04 for children <18 years and 2007–08 for adults >65 years) increased substantially after the adjustments, better highlighting the morbidity associated with influenza infections during those earlier seasons (Technical Appendix Table 3). The wide CIs in the adjusted rates for adults >65 years in all seasons reflects the poor sensitivity of influenza diagnostic tests in this age group.

**Figure 3:**
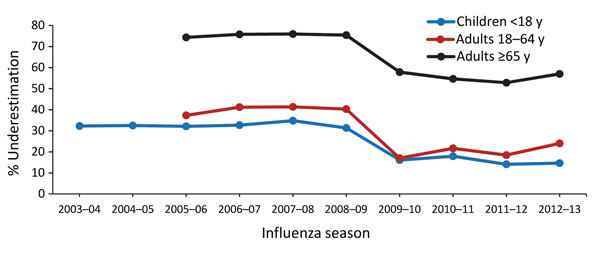
Underestimation of rates of influenza-associated hospitalization after adjustment for test sensitivity, by patient age group, Centers for Disease Control and Prevention Influenza Hospital Surveillance Network (FluSurv-NET), 2003–2013.

When adjusted for test sensitivity, observed rates of hospitalization underestimated influenza-associated hospitalization rates for all age groups but especially for adults >65 years (Figure 3). Observed hospitalization rates underestimated adjusted rates by ≈30% during 2003–2008 versus 15% during 2009–2013 for children <18 years; by 40% during 2005–2008 versus 20% during 2009–2013 for adults 18–64 years; and by 75% during 2005–2008 versus 55% during 2009–2013 for adults >65 years.

## Discussion

Adjusting for influenza diagnostic test sensitivity reveals that observed rates of influenza-associated hospitalization currently reported from surveillance data underestimate influenza-associated hospitalizations, particularly for adults >65 years. The increased use of high sensitivity tests, such as RT-PCR, after 2009 for all age groups has substantially reduced the degree of underestimation for children <18 years and adults 18–64 years of age. However, FluSurv-NET surveillance data still underestimate rates of influenza-associated hospitalization by 55% for adults >65 years without adjustments for influenza test sensitivity. Accurate influenza diagnostic testing can have a major impact on monitoring and guiding public health interventions for the control, prevention, and treatment of influenza.

Studies relying on administrative data alone to estimate rates of influenza-associated hospitalization may underestimate rates because influenza is seldom listed as a discharge diagnosis without laboratory-confirmed testing (*32*–*35*). The best way to ascertain influenza-associated hospitalization incidence rates in real time is to perform prospective surveillance that uses the most sensitive testing criteria (i.e., RT-PCR). Indeed, studies that have relied on active surveillance and testing, most often with RT-PCR, can improve estimates of influenza-associated hospitalization rates (*32*,*34*–*38*); however, as our study shows, failing to account for diagnostic test sensitivity can result in continued underestimation of influenza-associated hospitalizations, especially among older adults. Influenza diagnostic tests, regardless of test type, have poorer sensitivity in older adults than in younger persons. The methods used in our study account for case underascertainment resulting from varying testing sensitivity and provide opportunities to better compare the severity among different influenza seasons and age groups.

Although the degree of underestimation for the hospitalization rates reported here may seem high, the adjusted rates per 100,000 population for adults >65 years of age of 155.2 during 2005–06, 67.3 during 2006–07, and 314.4 during 2007–08 are still lower than the rates estimated in the literature using models of administrative data (rates per 100,000 for adults >65 years were 291.9 during 2005–06, 136.9 during 2006–07, and 380.9 during 2007–08) (*13*). This difference may be due to patients who had an influenza-associated hospitalization but were missed by our system because they were not tested. Nevertheless, sensitivity adjustments enable us to further improve the accuracy of estimated rates of influenza-associated hospitalization and provide timely results that account for changes in diagnostic test sensitivity over time.

Our analysis has limitations. First, our sensitivity adjustments do not reflect differences in detection by type or subtype of influenza viruses. Although this is a limitation of our analysis because diagnostic test sensitivity can vary on the basis of type or subtype of influenza viruses (*7*,*21*,*23*), differences in sensitivity based on type or subtype would have been difficult to assess, especially before the 2009 pandemic, when those data were not routinely available because of lack of RT-PCR or viral culture data in our network. Second, we did not adjust for any further variation in sensitivity measures by individual diagnostic test, but sensitivity measurements obtained from the literature enabled more generalizable estimates across the entire surveillance system. Third, we did not account for diagnostic test specificity. Influenza diagnostics tests generally have high specificities ranging from 96% to 100% regardless of age group (*7*,*10*,*11*), and the specificities of the tests used in the surveillance system have remained relatively constant over the study period, unlike test sensitivity. Although accounting for false-positive test results might decrease our estimates, the impact on overall rates would be minimal because test sensitivity covered much wider ranges. Fourth, although we conducted an extensive literature review, we did not conduct a formal systematic literature review. Additionally, published data on test sensitivity in adults >65 years of age are sparse; however, most studies demonstrate the poor sensitivity of influenza diagnostic tests in this particular population. Studies with larger sample sizes that focus on adults >65 years of age would improve understanding of diagnostic test sensitivity in this population with greater precision than is currently known. Finally, diagnostic testing in FluSurv-NET depends on a health care provider’s decision to order diagnostic testing on an individual patient. Therefore, we were unable to account for patients with influenza who were not tested. Multipliers based on the probability of an influenza-infected patient’s being tested have been estimated from the 2010–11 and 2011–12 seasons to correct for underascertainment (*39*; Technical Appendix). Rates were adjusted for diagnostic test sensitivity and frequency of influenza testing (Technical Appendix Table 3); however, because these results derive from estimates from 2 influenza seasons after the 2009 pandemic, our ability to determine whether the propensity to test has truly changed over time remains limited.

In conclusion, despite the increased use of highly sensitive molecular assays, current FluSurv-NET data still underestimate rates of influenza-associated hospitalization, particularly in adults >65 years of age. The primary reason for this underestimation is that diagnostic test sensitivity is imperfect, so true cases of influenza are missed. Furthermore, test sensitivity varies with patient age, and all types of influenza diagnostic tests, but especially RIDTs, have comparatively poor sensitivity in older persons. Adjusting hospitalization rates on the basis of diagnostic test sensitivity enables more accurate and timely comparisons of associated disease activity in hospitalized patients over time.

Technical Appendix FluSurv-NET coverage area and results, influenza diagnostic sensitivity range from literature review, and applied probability distribution by age group.
