# Supplementary material for: Improving Accuracy of Influenza-Associated Hospitalization Rate Estimates
Source: Emerg Infect Dis. 2015 Sep;21(9):1595–601. doi: 10.3201/eid2109.141665 (PMC4550134; doi:10.3201/eid2109.141665)
Supplement: Technical Appendix — FluSurv-NET coverage area and results, influenza diagnostic sensitivity range from literature review, and applied probability distribution by age group. [file 14-1665-Techapp-s1.pdf]

Article DOI: http://dx.doi.org/10.3201/eid2109.141665

Improving Accuracy of Influenza-Associated Hospitalization Rate Estimates

Technical Appendix

FluSurv-NET Coverage Area

FluSurv-NET covers ≈70 counties in the 10 Emerging Infections Program states (California, Colorado, Connecticut, Georgia, Maryland, Minnesota, New Mexico, New York, Oregon, and Tennessee). Since the 2009 influenza pandemic, the network expanded to include counties with defined catchment-area populations from other states: Iowa (2009–10, 2012–13 seasons), Idaho (2009–10, 2010–11 seasons), Michigan (2009–10 through 2012–13 seasons), Ohio (2010–11 through 2012–13 seasons), Oklahoma (2009–10, 2010–11 seasons), Rhode Island (2010–11 through 2012–13 seasons), South Dakota (2009–10 season), and Utah (2010–11 through 2012–13 seasons).

Results

During 2003–2013, the distribution of types of influenza diagnostic tests among identified cases changed, particularly after the 2009 pandemic, for each age group. Before the 2009 pandemic, rapid tests were the most common test types among identified cases in FluSurv-NET (Technical Appendix Figure). After the 2009 pandemic, reverse transcription PCR (RT-PCR) became the most common test type among identified cases in all age groups.

Technical Appendix Table 1 shows influenza diagnostic test sensitivity ranges from the literature review and applied probability distribution by age group.

Technical Appendix Table 2 shows overall FluSurv-NET observed and adjusted rates of hospitalization per 100,000 population during 2003–2013 for all age groups.

Technical Appendix Table 3 shows FluSurv-NET observed rates of hospitalization per 100,000 population; hospitalization rates adjusted for test sensitivity; and hospitalization rates adjusted for test sensitivity and frequency of testing by patient age group during 2003–2013.

Adjustments attempting to account for frequency of testing have previously been performed by using the following methods. The frequency of influenza testing among hospitalized patients with respiratory infections was assessed in a sample of 5 participating surveillance areas during December–April of the 2010–11 and 2011–12 seasons. Sites selected hospitals that were representative of their catchment area and identified all patients who had been admitted with respiratory infections by using a discharge audit of International Classification of Diseases, Ninth Revision, codes 466–488. For an age-stratified random sample, the proportion of patients tested for influenza was assessed from review of medical charts and laboratory records (*19*). The proportion of patients tested for influenza was 63.8% (53.8%–73.8%) for children <18 years of age, 39.6% (30.4%–48.8%) for adults 18–64 years, and 29.4% (22.3%–36.5%) for adults ≥65 years. The adjustments for testing frequency was extrapolated from data derived from 2 influenza seasons after the 2009 pandemic. Testing frequency data from before the pandemic are not available, and clinician testing practices before 2009 might have differed from the practices after the pandemic. After adjustment for sensitivity and frequency of testing, observed rates of hospitalization underestimated adjusted rates by ≈57% during 2003–2009 versus ≈46% during 2009–2013 for children <18 years of age, by ≈76% during 2005–2008 versus ≈68% during 2009–2013 for adults 18–64 years, and by ≈93% during 2005–2008 versus ≈87% during 2009–2013 for adults ≥65 years.

Technical Appendix Table 1. Influenza diagnostic test sensitivity range from literature review and applied probability distribution by age group, FluSurv-NET, 2003–2013

Diagnostic test/patient age group, y	Mid-estimate, %	Sensitivity range, %	Probability distribution	Reference
RT-PCR				
0–17	–	79.2–100.0	Uniform	(*1*)
0–17	–	85.0–98.4	Uniform	(*2*)
0–17	–	95.3–100.0	Uniform	(*2*)
0–17	–	88.4–100.0	Uniform	(*2,3*)
0–17	–	93–98.4	Uniform	(*4,5*)
18–64	–	79.2–100.0	Uniform	(*1*)
18–64	–	85.0–98.4	Uniform	(*2*)
18–64	–	95.3–100.0	Uniform	(*3*)
18–64	–	88.4–100.0	Uniform	(*2,3,5*)
≥65	–	79.2–93.0	Uniform	(*1,6,7*)
Culture				
0–17	–	56.0–100.0	Uniform	(*7*)
0–17	–	45.0–89.0	Uniform	(*5,9*)
0–17	–	60.3–75.0	Uniform	(*10*)
18–64	–	56.0–100.0	Uniform	(*8*)
18–64	–	45.0–89.0	Uniform	(*5,9*)

Diagnostic test/patient age group, y	Mid-estimate, %	Sensitivity range, %	Probability distribution	Reference
≥65	–	19.4–53.8	Uniform	(*7,11*)
DFA	–			
0–17	–	76.7–78.4	Uniform	(*4*)
0–17	62.0	45.0–65.0	Normal	(*12*)
0–17	73.9	59.8–90.0	Normal	(*12*)
0–17	–	45.0–90.0	Uniform	(*13*)
0–17	–	53.0–84.2	Uniform	(*14,15*)
18–64	–	53.0–84.2	Uniform	(*14,15*)
≥65	–	53.0–84.2	Uniform	(*14,15*)
RIDT				
0–17	66.6	61.6–71.7	Normal	(*16*)
18–64	53.9	47.9–59.8	Normal	(*16*)
≥65	–	8.5–43.0	Uniform	(*7,11,17*)
≥65	–	8.0–28.0	Uniform	(*18*)

*DFA, direct fluorescence antibody; RIDT, rapid influenza diagnostic test; RT-PCR, reverse transcription PCR; –, no mid-estimates from the literature review.

Technical Appendix Table 2. FluSurv-NET hospitalization rates per 100,000 population, 2003–2013

Influenza season	Observed rate (95% CI)	Rate adjusted for test sensitivity (95% CI)
2003–04*	30.6 (28.9–32.3)	45.2 (41.0–50.4)
2004–05*	13.5 (12.4–14.6)	20.0 (17.8–23.1)
2005–06	15.0 (4.2–42.1)	22.0 (6.8–308.5)
2006–07	10.1 (2.8–17.6)	15.0 (4.6–137.2)
2007–08	16.3 (9.1–78.4)	24.0 (15.0–602.0)
2008–09	13.6 (3.8–15.9)	22.0 (6.1–110.1)
2009–10	30.6 (28.6–51.7)	59.0 (34.7–123.3)
2010–11	20.2 (14.1–66.7)	24.7 (17.6–234.3)
2011–12	7.3 (5.0–31.5)	8.5 (6.1–102.5)
2012–13	28.5 (22.9–185.3)	33.6 (29.3–731.9)

*Data are based only on estimate from children <18 y.

Technical Appendix Table 3. FluSurv-NET hospitalizations rates per 100,000 population, 2003–2013*

Age group, y/influenza season	Observed rate (95% CI)	Rate adjusted for test sensitivity (95% CI)	Rate adjusted for test sensitivity and frequency of testing†
Children <18			
2003–04	30.6 (28.9–32.3)	45.2 (41.0–50.4)	70.8 (55.6–93.7)
2004–05	13.5 (12.4–14.6)	20.0 (17.8–23.1)	31.3 (24.1–42.9)
2005–06	15 (13.9–16.1)	22.2 (19.7–25.4)	34.5 (26.7–47.2)
2006–07	10.1 (9.2–11.0)	15.0 (13.2–17.0)	23.5 (17.9–31.6)
2007–08	16.3 (15.2–17.5)	25.0 (21.7–26.7)	39.2 (29.4–49.6)
2008–09	15.1 (14.0–16.1)	22.0 (19.7–24.6)	34.5 (26.7–45.7)
2009–10	50.5 (48.6–52.3)	60.2 (56.8–66.4)	94.4 (77.0–123.4)
2010–11	20.2 (19.2–21.3)	24.6 (22.7–27.4)	38.6 (30.8–50.9)
2011–12	7.3 (6.7–8.0)	8.5 (7.7–9.8)	13.3 (10.4–18.2)
2012–13	28.5 (27.1–29.7)	33.4 (31.2–37.3)	52.4 (42.3–69.3)
Adults 18–64			
2003–04	–	–	–
2004–05	–	–	–
2005–06	4.7 (4.1–4.9)	7.5 (6.6–8.6)	18.9 (13.5–28.3)
2006–07	3.0 (2.7–3.3)	5.1 (4.4–5.9)	12.9 (9.0–19.4)
2007–08	9.5 (9.0–10.0)	16.2 (14.7–18.3)	40.9 (30.1–60.1)
2008–09	4.0 (3.7–4.4)	6.7 (5.9–7.6)	16.9 (12.1–25.0)
2009–10	30.3 (29.5–31.2)	36.5 (34.3–40.8)	92.2 (70.3–134.1)
2010–11	14.5 (14–15.1)	18.5 (17.3–20.5)	46.7 (35.5–67.4)
2011–12	5.3 (5–5.7)	6.5 (6.0–7.3)	16.4 (12.3–24.0)
2012–13	23.4 (22.7–24)	30.8 (28.8–33.9)	77.8 (78.9–111.4)
Adults ≥65			
2003–04	–	–	–
2004–05	–	–	–
2005–06	39.9 (37–42.9)	155.2 (89.3–359.8)	527.9 (244.7–1613.4)
2006–07	16.3 (14.7–18.0)	67.3 (37.6–154.8)	228.9 (103.0–694.2)
2007–08	75.8 (72.4–79.4)	314.4 (172.8–686.7)	1069.4 (473.4–3079.3)
2008–09	13.6 (12.2–15.3)	55.3 (29.5–123.8)	188.1 (80.8–555.2)
2009–10	30.0 (27.9–32)	71.1 (47.7–138.5)	241.8 (130.7–621.1)
2010–11	64.7 (62.1–67.6)	142.5 (98.9–256.4)	484.7 (271.0–1149.8)
2011–12	30.1 (28.3–32.1)	63.8 (44.8–114.6)	217.0 (122.7–513.9)

Age group, y/influenza season	Observed rate (95% CI)	Rate adjusted for test sensitivity (95% CI)	Rate adjusted for test sensitivity and frequency of testing†
2012–13	181.8 (177.3–186.4)	422.6 (286.6–803.6)	1437.4 (785.2–3598.7)

*–, No data on adults were collected until the 2005–06 influenza season
†Frequency of influenza estimating among hospitalized patients with respiratory infections is based on data from the 2010–11 and 2011–12 influenza seasons. Those frequencies are 63.8% (53.8%–73.8%) for children <18 y; 39.6% (30.4%–48.8%) for adults 18–64 y; and 29.4% (22.3%–36.5%) for adults ≥65 y (*19*).

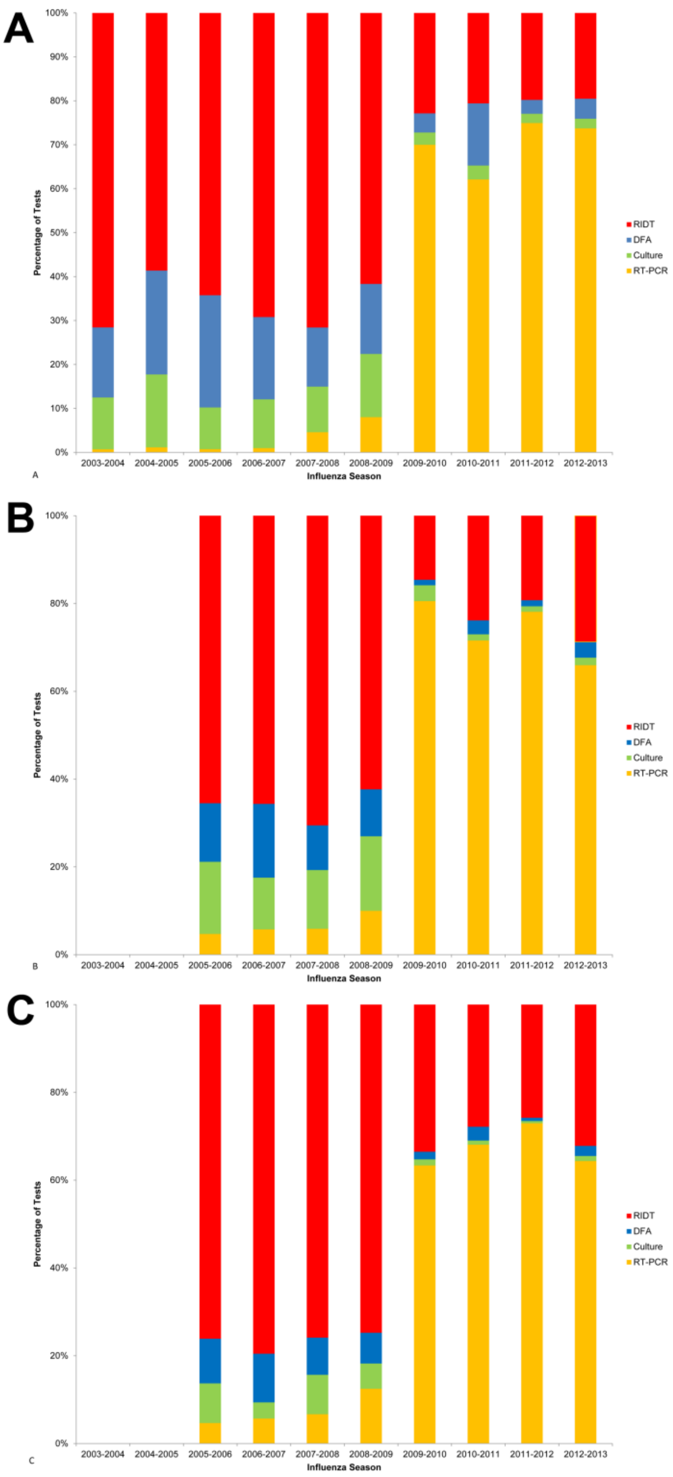

Technical Appendix Figure. Distribution of influenza diagnostic tests among identified cases, by patient age group, in FluSurv-NET, 2003–2013. DFA, direct fluoresce antibody; RIDT, rapid influenza diagnostic test; RT-PCR, reverse transcription PCR. A) Children <18 years of age. B) Adults 18–64 years of age. C) Adults $\geq$65 years of age.

References

1. Novak-Weekley SM, Marlowe EM, Poulter M, Dwyer D, Speers D, Rawlinson W, et al. Evaluation of the Cepheid Xpert Flu Assay for rapid identification and differentiation of influenza A, influenza A 2009 H1N1, and influenza B viruses. J Clin Microbiol. 2012;50:1704–10. PubMed http://dx.doi.org/10.1128/JCM.06520-11

2. Van Wesenbeeck L, Meeuws H, Van Immerseel A, Ispas G, Schmidt K, Houspie L, et al. Comparison of the FilmArray RP, Verigene RV+, and Prodesse ProFLU+/FAST+ multiplex platforms for detection of influenza viruses in clinical samples from the 2011–2012 influenza season in Belgium. J Clin Microbiol. 2013;51:2977–85. PubMed http://dx.doi.org/10.1128/JCM.00911-13

3. Templeton KE, Scheltinga SA, Beersma MF, Kroes AC, Claas EC. Rapid and sensitive method using multiplex real-time PCR for diagnosis of infections by influenza A and influenza B viruses, respiratory syncytial virus, and parainfluenza viruses 1, 2, 3, and 4. J Clin Microbiol. 2004;42:1564–9. PubMed http://dx.doi.org/10.1128/JCM.42.4.1564-1569.2004

4. Gharabaghi F, Tellier R, Cheung R, Collins C, Broukhanski G, Drews SJ, et al. Comparison of a commercial qualitative real-time RT-PCR kit with direct immunofluorescence assay (DFA) and cell culture for detection of influenza A and B in children. J Clin Virol. 2008;42:190–3. PubMed http://dx.doi.org/10.1016/j.jcv.2008.01.013

5. Steininger C, Kundi M, Aberle SW, Aberle JH, Popow-Kraupp T. Effectiveness of reverse transcription–PCR, virus isolation, and enzyme-linked immunosorbent assay for diagnosis of influenza A virus infection in different age groups. J Clin Microbiol. 2002;40:2051–6. PubMed http://dx.doi.org/10.1128/JCM.40.6.2051-2056.2002

6. Shetty AK, Treynor E, Hill DW, Gutierrez KM, Warford A, Baron EJ. Comparison of conventional viral cultures with direct fluorescent antibody stains for diagnosis of community-acquired respiratory virus infections in hospitalized children. Pediatr Infect Dis J. 2003;22:789–94. PubMed http://dx.doi.org/10.1097/01.inf.0000083823.43526.97

7. Walsh EE, Cox C, Falsey AR. Clinical features of influenza A virus infection in older hospitalized persons. J Am Geriatr Soc. 2002;50:1498–503. PubMed http://dx.doi.org/10.1046/j.1532-5415.2002.50404.x

8. Takahashi H, Otsuka Y, Patterson BK. Diagnostic tests for influenza and other respiratory viruses: determining performance specifications based on clinical setting. J Infect Chemother. 2010;16:155–61. PubMed http://dx.doi.org/10.1007/s10156-010-0035-Y

9. LaSala PR, Bufton KK, Ismail N, Smith MB. Prospective comparison of R-mix™ shell vial system with direct antigen tests and conventional cell culture for respiratory virus detection. J Clin Virol. 2007;38:210–6. PubMed http://dx.doi.org/10.1016/j.jcv.2006.12.015

10. Gharabaghi F, Hawan A, Drews SJ, Richardson SE. Evaluation of multiple commercial molecular and conventional diagnostic assays for the detection of respiratory viruses in children. Clin Microbiol Infect. 2011;17:1900–6. PubMed http://dx.doi.org/10.1111/j.1469-0691.2011.03529.x

11. Talbot HK, Williams JV, Zhu Y, Poehling KA, Griffin MR, Edwards KM. Failure of routine diagnostic methods to detect influenza in hospitalized older adults. Infect Control Hosp Epidemiol. 2010;31:683–8. PubMed http://dx.doi.org/10.1086/653202

12. Uyeki TM. Influenza diagnosis and treatment in children: a review of studies on clinically useful tests and antiviral treatment for influenza. Pediatr Infect Dis J. 2003;22:164–77. PubMed http://dx.doi.org/10.1097/01.inf.0000050458.35010.b6

13. Stout C, Murphy MD, Lawrence S, Julian S. Evaluation of a monoclonal antibody pool for rapid diagnosis of respiratory viral infections. J Clin Microbiol. 1989;27:448–52. PubMed

14. Rahman M, Kieke BA, Vandermause MF, Mitchell PD, Greenlee RT, Belongia EA. Performance of Directigen flu A+B enzyme immunoassay and direct fluorescent assay for detection of influenza infection during the 2004–2005 season. Diagn Microbiol Infect Dis. 2007;58:413–8. PubMed http://dx.doi.org/10.1016/j.diagmicrobio.2007.03.011

15. Irmen KE, Kelleher JJ. Use of monoclonal antibodies for rapid diagnosis of respiratory viruses in a community hospital. Clin Diagn Lab Immunol. 2000;7:396–403. PubMed

16. Chartrand C, Leeflang MM, Minion J, Brewer T, Pai M. Accuracy of rapid influenza diagnostic tests: a meta-analysis. Ann Intern Med. 2012;156:500–11. PubMed http://dx.doi.org/10.7326/0003-4819-156-7-201204030-00403

17. Fader RC. Comparison of the Binax NOW Flu A enzyme immunochromatographic assay and R-Mix shell vial culture for the 2003–2044 influenza seasons. J Clin Microbiol. 2005;43:6133–5. PubMed http://dx.doi.org/10.1128/JCM.43.12.6133-6135.2005

18. Steininger C, Redlberger M, Graninger W, Kundi M, Popow-Kraupp T. Near-patient assays for diagnosis of influenza virus infection in adult patients. Clin Microbiol Infect. 2009;15:267–73. PubMed http://dx.doi.org/10.1111/j.1469-0691.2008.02674.x

19. Reed C, Chaves SS, Daily Kirley P, Emerson R, Aragon D, Hancock EB, et al. Estimating influenza disease burden from population-based surveillance data in the United States. PLoS ONE. 2015;10:e0118369. PubMed http://dx.doi.org/10.1371/journal.pone.0118369
